# Hypertensive Urgency Secondary to a Malignant Pheochromocytoma and Its Complex Treatment Course: A Case Report

**DOI:** 10.7759/cureus.28702

**Published:** 2022-09-02

**Authors:** Amr Elkammash, Mustafa Alsinan, Khaled Madi, Ahmed Abbas, Nourhan Degheidy

**Affiliations:** 1 Cardiology, The Royal Papworth Hospital NHS Foundation Trust, Cambridge, GBR; 2 Internal Medicine, Princess of Wales Hospital, Swansea, GBR; 3 Internal Medicine, University Hospitals Dorset NHS Foundation Trust, The Royal Bournemouth Hospital, Bournemouth, GBR; 4 Internal Medicine, Sunderland Royal Hospital, Sunderland, GBR; 5 Chemical Pathology, The Medical Research Institute, Alexandria, EGY

**Keywords:** palliative care, high blood pressure, acute limb ischaemia, complications, chemotherapy, metastasis, malignant, pheochromocytoma

## Abstract

Pheochromocytoma originates from the chromaffin cells of the adrenal medulla. It produces an excess of catecholamines. It is essentially a benign tumour, and the malignant type represents a minority. The malignant behaviour can be unclear in the absence of metastases. Factors of poor prognosis in malignant pheochromocytoma include male sex, old age, large-sized tumours, the presence of metastases at the time of diagnosis and non-surgical treatment. The cornerstone treatment of pheochromocytoma is surgical excision. In the presence of metastases, chemotherapy can control the symptoms and prolong survival. Its reported side effects are usually few and mild.

This report presents a rare case of malignant pheochromocytoma in a 26-year-old gentleman that first manifested as a hypertensive urgency. The patient had several recurrences and multiple metastases despite two surgical excisions. Such poor outcome could not be predicted initially by the known risk factors. A non-previously reported complication of treatment was acute lower limb ischaemia after the start of chemotherapy for the tumour, depriving the patient of completing the course.

In conclusion, the presence of hypertension in young adults warrants the investigation for pheochromocytoma. Postoperative follow-up is mandatory to pick up early signs of malignancy and metastasis. Tumour breakdown by chemotherapy can cause various cardiovascular problems including acute limb ischaemia. The management can be quite challenging, therefore, a multidisciplinary team should look after the case. A palliative approach can be used in patients with severe symptoms and no chance of cure.

## Introduction

Pheochromocytomas are a rare cause of secondary hypertension. They form 0.2-0.6% of such cases [1]. They originate from the chromaffin cells of the adrenal medulla and secrete excess catecholamines (norepinephrine, epinephrine and dopamine). They are essentially benign tumours, with only 2-15% being malignant [2]. The malignant type of pheochromocytoma is only diagnosed in the presence of extra-chromaffin spread. The prognosis of the tumour is unpredictable at the initial presentation. Researchers have postulated predictive factors for tumour aggressiveness including large tumour size, male sex, old age, synchronous metastases and non-surgical management [3]. Surgical excision is the main line of treatment. Chemotherapy improves symptoms and survival in patients with metastatic disease [4]. However, there are scanty reports on the side effects of chemotherapy in pheochromocytoma.

We report a case of hypertensive urgency caused by a malignant pheochromocytoma, with multiple recurrences despite repeated surgical excisions. Chemotherapy of the tumour was complicated with acute lower limb ischaemia. We also tested the effectiveness of different laboratory markers in the diagnosis of tumour recurrence and the performance of the published prognostic factors in the prediction of tumour behaviour.

## Case presentation

**Figure 1:**
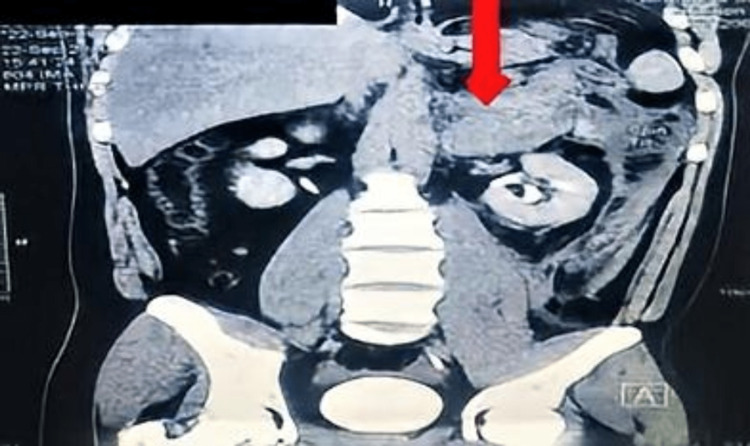
Multiphasic CT of adrenal glands coronal section showing a left adrenal heterogenous mass pushing the left kidney downwards (arrow).

**Figure 2:**
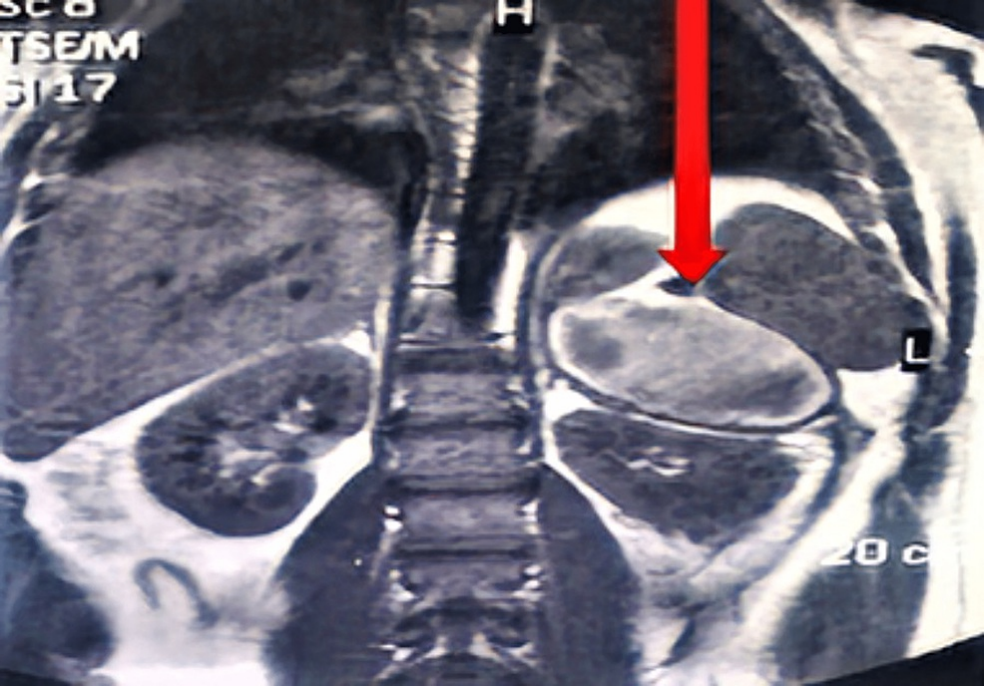
MRI abdomen with adrenal protocol coronal view, showing a heterogenous left adrenal tumour overlying the upper pole of the left kidney (arrow).

A 26-year-old male presented to the emergency department with severe headache, palpitations and heavy sweating. He suffered from recurrent severe frontal headache and dull aching left loin pain for five years before presentation. His general practitioner said his blood pressure (BP) was quite high and could not be controlled with a single antihypertensive medication (bisoprolol 5 mg once daily). On examination, he was heavily sweaty. His BP was 200/140 mmHg bilaterally. We neither found a significant difference between the upper and lower limbs nor a radio-femoral delay. The pulse was 120 beats per minute and bounding in character. The heart sounds were normal. The lungs were clear to auscultation. There was a firm left lumbar mass on abdominal palpation that moved with respiration. The fundus examination revealed grade 2 hypertensive retinopathy. The patient was admitted to the cardiac care unit as a case of hypertensive urgency. He was started on intravenous labetalol infusion with a sub-optimal control of BP. The laboratory workup showed a raised 24 hours urinary metanephrines of 1078.6 µg/24 hours (the normal reference value was less than 1000 µg/24 hours). An abdominal ultrasound showed a heterogeneous soft tissue mass related to the upper pole of the left kidney. A multi-phasic CT scan of the adrenal glands showed a 4.5x7.5x5.5 cm heterogeneous left adrenal mass, mild free peritoneal collection, stranding of related fat planes and thickening of the left diaphragmatic crus (Figure 1). An MRI scan of the abdomen with adrenal protocol confirmed the presence of a 6.5x4x5 cm left adrenal tumour, T1 isointense and T2 hyperintense, associated with a posterior subacute hematoma (Figure 2).

The patient went for a whole body CT scan with contrast that ruled out the presence of metastases. A multidisciplinary team (MDT) discussed the case, including a cardiologist, an endocrinologist, a urologist, a radiologist and an anaesthetist. The team decided on the excision of the tumour after a good pharmacological preparation, and the patient underwent laparoscopic left adrenalectomy. The microscopic assessment of the excised tumour revealed tumour cells arranged in well-defined nests bounded by a delicate fibrovascular stroma. The cells have indistinct cell boundaries and granular cytoplasm. The nuclei are oval with prominent nucleoli. Wide areas of chronic inflammatory cells (lymphocytes, plasma cells, hemosiderin-laden macrophages) were detected. This picture confirmed the diagnosis of pheochromocytoma. We could stop all the antihypertensive medications with the stability of the BP. We checked the plasma metanephrines one month after the surgery and they were normal (the normetanephrine was 90 pg/ml (normal level is 18-111 pg/ml) and the metanephrine was 40 pg/ml (normal level is 12-60 pg/ml)). A surveillance CT scan of the abdomen one year after the surgery did not show any recurrence, with normal levels of serum metanephrines. Therefore, the MDT decided to follow a two-yearly surveillance protocol according to the local guidelines.

**Figure 3:**
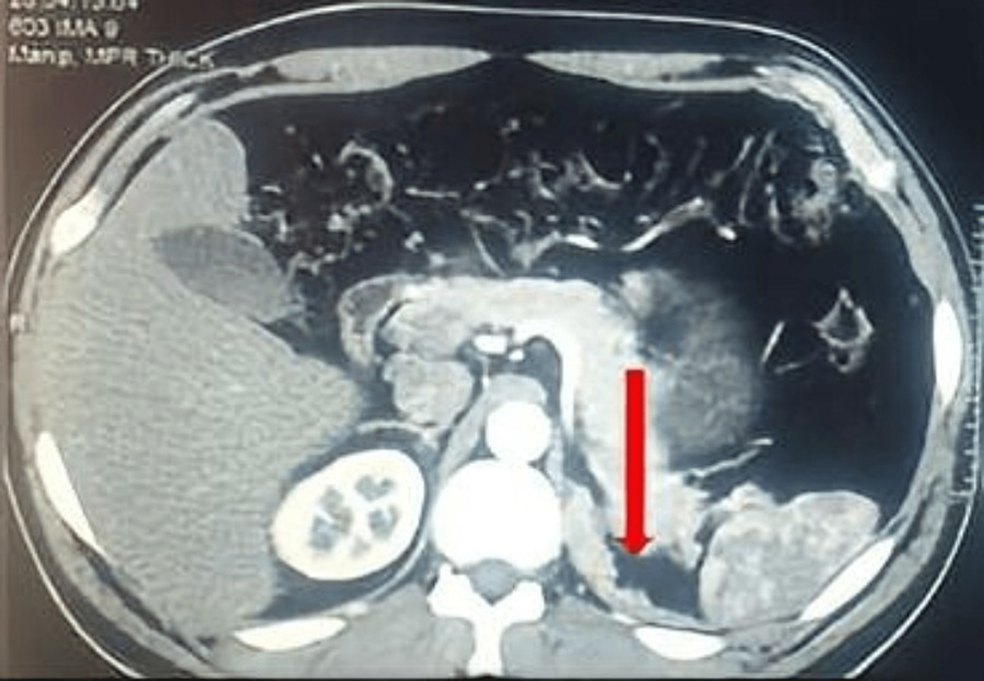
Multiphasic CT of the adrenals transverse view, showing a new irregular tumour growth at the site of the previous tumour excision (arrow).

A year later, the patient complained of recurrence of the headaches and the left loin pain. He also felt shortness of breath and fast palpitations of minimal exertion. A 24-hour urinary vanillyl mandelic acid (VMA) was raised (18.7 mg/day; the normal reference value was less than 13.6 mg/day). A multiphasic CT of adrenal glands showed an irregular soft tissue lesion along the left diaphragmatic and posterior pararenal space measuring 4x2x9 cm (Figure 3). A repeat full body CT scan with contrast did not show metastases. The case was presented again to the MDT and they decided to perform a second open excision for the recurred tumour with a wider safety margin. The patient went to the second operation after a detailed discussion about the expected higher complication risk. The pathological examination of the excised tissue confirmed the recurrence of the pheochromocytoma.

**Figure 4:**
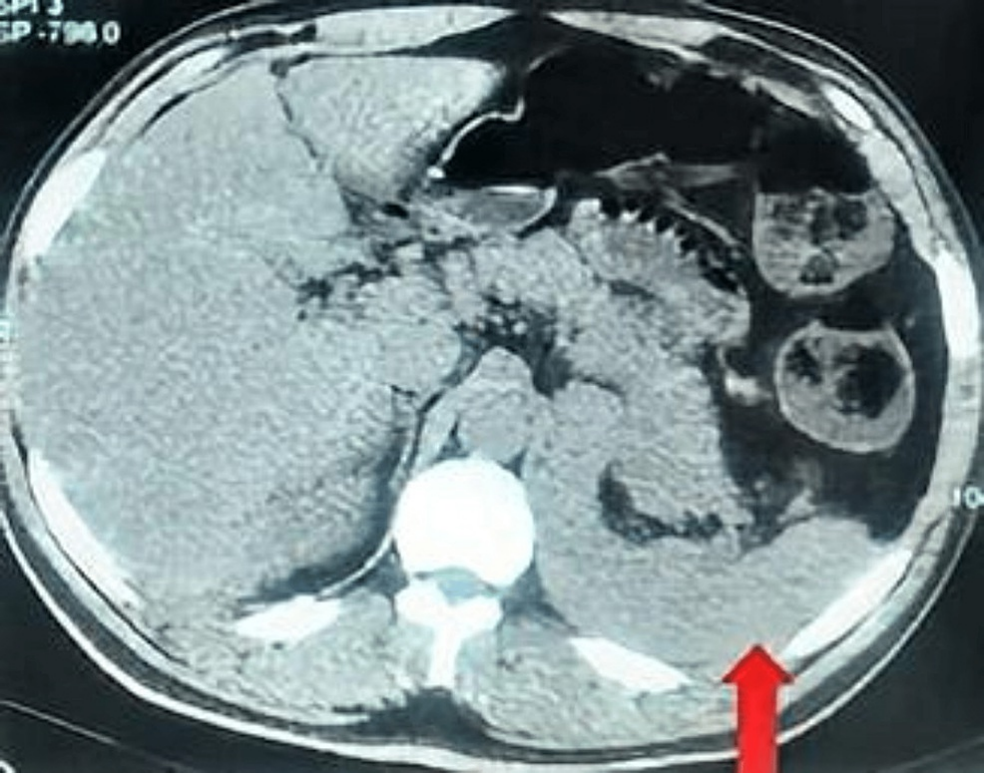
A contrast CT abdomen transverse view showing a recurrence of the tumour in the left adrenal bed (arrow).

The one-week postoperative levels of serum metanephrines were normal. The patient remained asymptomatic for six months. He then started to experience a recurrence of his headaches, palpitations and shortness of breath. His 24-hour urinary metanephrine was markedly raised above 4000 mg/day (the normal reference range was 25-312 mg/day), and his 24-hour urinary VMA was within normal (5.3 mg/day; the normal reference value was 1.5-6.5 mg/day). A whole body CT scan with contrast showed a left retroperitoneal mass 15x11x5 cm displacing the left kidney and the spleen anteriorly and abutting the left diaphragmatic crus associated with new peritoneal and lung nodules (Figure 4).

**Figure 5:**
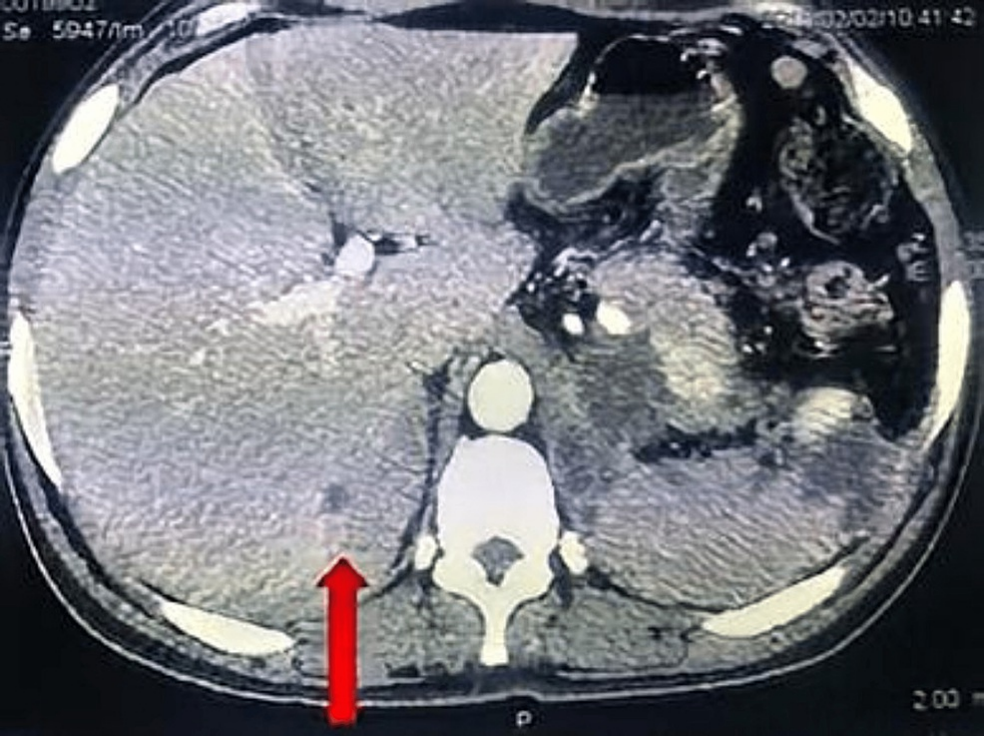
A contrast CT abdomen transverse view showing new liver metastasis (arrow).

A medical oncology consultant was involved in the multidisciplinary discussion, and he planned to give the patient four cycles of chemotherapy (cyclophosphamide, vincristine and dacarbazine; CVD) to control the patient’s symptoms and the tumour spread. The patient complained of acute left foot and leg pain after the third cycle of chemotherapy. On examination, his leg and foot were quite pale and cold with early peripheral cyanosis in the toes. The posterior tibial and dorsalis pedis pulses were lost. A vascular surgeon reviewed the patient urgently and he concluded the diagnosis of an acute limb ischaemia. This was confirmed by an enhanced CT angiogram of the lower limbs revealing segmental narrowing in the left anterior and posterior tibial arteries without thrombotic occlusion. The patient was immediately started on IV alprostadil with oral cilostazol and amlodipine. His symptoms improved quickly after starting him on the vasodilators and a repeat CT angiogram confirmed the restoration of the normal vessels' caliber. We gradually weaned off the medications over four weeks without recurrence of symptoms. That event prevented him from completing the course of chemotherapy. A follow-up laboratory work-up showed raised serum chromogranin A=234.8 ng/ml (the normal reference value was up to 100 ng/ml), 24-hour urinary metanephrines more than 4000 mg/day (the normal reference range was 25-312 mg/day); and the urinalysis showed a 24-hour urinary protein 221 mg/day, signifying microalbuminuria and hypertensive nephropathy. A follow-up contrast-enhanced whole-body CT five months later revealed an increase in the size of the tumour (6.5x13x19.4 cm), with spreading to the liver, para-aortic lymph nodes and peritoneum (Figure 5).

The patient’s symptoms continued to deteriorate; therefore, he was referred to the palliative care team for symptomatic management.

## Discussion

The reported case represents a hypertensive urgency secondary to a rare malignant type of pheochromocytoma. The tumour was quite aggressive, with three recurrences in two and half years. Acute lower limb ischaemia complicated the chemotherapy course. The palliative approach seemed the last resort to control the patient's symptoms.

The presence of metastases carries a poor prognosis and high mortality [3]. Chemotherapy with CVD showed symptomatic and survival benefits in patients with metastatic pheochromocytoma [5]. It can release catecholamines from tumour cell breakdown with various cardiovascular complications [4]. Acute vasospastic limb ischaemia is not a known side effect of the chemotherapy used in this case [6]. The patient did not have any other risk factors for peripheral vascular disease. This indicates that the perceived complication is due to tumour lysis. A case of hypertension induced by CVD protocol was previously reported [7]. To our knowledge, we are the first group to report acute lower limb ischaemia caused by CVD chemotherapy. This may have future implications on the clinical care and follow-up on pheochromocytoma patients on systemic chemotherapy.

Our report demonstrates the usefulness of different laboratory markers in the diagnosis of pheochromocytoma. We used urinary VMA, urinary metanephrines and serum chromogranin A. Urinary metanephrines were more accurate than urinary VMA in the diagnosis of the second recurrence in our patient. This is reflected in the recommendations of the European Society of Endocrinology guidelines on the use of urinary metanephrines for post-operative follow-up of pheochromocytoma patients [8].

Researchers found that the aggressiveness of pheochromocytoma can be predicted by male sex, older age (more than 50 years old), larger tumour size (more than 10 cm), the presence of simultaneous metastases at the time of diagnosis and non-surgical treatment strategy [3]. None of these factors, except for the male sex, predicted the aggressiveness of the tumour in our case.

The limitations to our case management were the non-use of radioactive iodine-labelled metaiodobenzylguanidine (^123^I-MIBG) scintigraphy and/or PET CT scan to look for metastases and the non-performance of genetic testing on the patient and his family. These should be considered in the management of patients with metastatic pheochromocytoma [9].

## Conclusions

In conclusion, malignant pheochromocytoma is an extremely rare cause of secondary hypertension. It should be considered in the assessment of hypertension in young adults. It is difficult to predict the aggressiveness of such a tumour. A multidisciplinary approach should be adopted in its management. Surgical resection remains the mainstay treatment. A close post-operative follow-up is mandatory to detect early recurrence and distant spread. Chemotherapy can provide symptoms and survival benefits; however, it can lead to cardiovascular complications secondary to tumour breakdown and massive catecholamine release. A palliative approach can be followed in uncontrollable cases.

## References

[1] Shah NH, Ruan DT (2014). Pheochromocytoma: a devious opponent in a game of hide-and-seek. Circulation.

[2] Aygun N, Uludag M (2020). Pheochromocytoma and paraganglioma: from epidemiology to clinical findings. Sisli Etfal Hastan Tip Bul.

[3] Hamidi O, Young WF Jr, Iñiguez-Ariza NM (2017). Malignant pheochromocytoma and paraganglioma: 272 patients over 55 years. J Clin Endocrinol Metab.

[4] Adjallé R, Plouin PF, Pacak K, Lehnert H (2009). Treatment of malignant pheochromocytoma. Horm Metab Res.

[5] Ayala-Ramirez M, Feng L, Habra MA (2012). Clinical benefits of systemic chemotherapy for patients with metastatic pheochromocytomas or sympathetic extra-adrenal paragangliomas: insights from the largest single-institutional experience. Cancer.

[6] Chang HM, Moudgil R, Scarabelli T, Okwuosa TM, Yeh ET (2017). Cardiovascular complications of cancer therapy: best practices in diagnosis, prevention, and management: part 1. J Am Coll Cardiol.

[7] Keiser HR, Goldstein DS, Wade JL, Douglas FL, Averbuch SD (1985). Treatment of malignant pheochromocytoma with combination chemotherapy. Hypertension.

[8] Plouin PF, Amar L, Dekkers OM (2016). European Society of Endocrinology Clinical Practice Guideline for long-term follow-up of patients operated on for a phaeochromocytoma or a paraganglioma. Eur J Endocrinol.

[9] Lenders JW, Duh QY, Eisenhofer G (2014). Pheochromocytoma and paraganglioma: an endocrine society clinical practice guideline. J Clin Endocrinol Metab.

